# Predicting Kyasanur forest disease in resource-limited settings using event-based surveillance and transfer learning

**DOI:** 10.1038/s41598-023-38074-0

**Published:** 2023-07-08

**Authors:** Ravikiran Keshavamurthy, Lauren E. Charles

**Affiliations:** 1 Pacific Northwest National Laboratory, Richland, WA 99354 USA; 2 Paul G. Allen School for Global Health, Washington State University, Pullman, WA 99164 USA

**Keywords:** Epidemiology, Infectious diseases, Viral infection

## Abstract

In recent years, the reports of Kyasanur forest disease (KFD) breaking endemic barriers by spreading to new regions and crossing state boundaries is alarming. Effective disease surveillance and reporting systems are lacking for this emerging zoonosis, hence hindering control and prevention efforts. We compared time-series models using weather data with and without Event-Based Surveillance (EBS) information, i.e., news media reports and internet search trends, to predict monthly KFD cases in humans. We fitted Extreme Gradient Boosting (XGB) and Long Short Term Memory models at the national and regional levels. We utilized the rich epidemiological data from endemic regions by applying Transfer Learning (TL) techniques to predict KFD cases in new outbreak regions where disease surveillance information was scarce. Overall, the inclusion of EBS data, in addition to the weather data, substantially increased the prediction performance across all models. The XGB method produced the best predictions at the national and regional levels. The TL techniques outperformed baseline models in predicting KFD in new outbreak regions. Novel sources of data and advanced machine-learning approaches, e.g., EBS and TL, show great potential towards increasing disease prediction capabilities in data-scarce scenarios and/or resource-limited settings, for better-informed decisions in the face of emerging zoonotic threats.

## Introduction

Rapidly changing environments along with increased interactions between humans, wildlife, and domesticated animals have resulted in a substantial and rising threat of emerging zoonotic diseases worldwide^1,2^. The knowledge of underlying mechanisms for their emergence is often unclear, hence predicting their spatial and temporal occurrence is challenging. Kyasanur forest disease (KFD) is one such zoonosis that is breaching endemic barriers and causing large outbreaks in new locations. The disease was first reported in the Kyasanur forest of Shimoga Districts, Karnataka, India in the year 1957^3^. Historically, KFD has remained endemic to Shimoga and its adjoining districts in Karnataka. However, over the past 15 years, frequent outbreaks outside the endemic foci have caused serious public health concerns. Between 2012 and 2014, the first major outbreaks of KFD outside the endemic area were reported in the Chamarajanagar district of Karnataka and the neighboring Malappuram and Wayanad districts of Kerala^4–6^. Similarly, in 2015 and 2016, disease outbreaks were reported in North Goa and Sindhudurg districts in Goa and Maharashtra, respectively^7,8^. The disease disproportionately affects poor and underprivileged communities living in or near forest areas. Despite its zoonotic and public health importance, the disease has been historically understudied and the reasons for its emergence have been largely unknown^9,10^.

The KFD is a tick-borne hemorrhagic fever caused by a virus belonging to the family *Flaviviridae*. The virus is maintained by ticks, mammals, and birds in the sylvatic life cycle^11^ and causes high mortalities in nonhuman primates, such as the black-faced langur (*Semnopithecus entellus*) and the red-faced bonnet monkey (*Macaca radiata*)^12,13^. KFD virus transmission occurs commonly through the bite of an infected *Haemaphysalis spinigera* and other related species of ticks^12,14^. The climatic variables, including temperature and precipitation-related parameters, are known to affect the KFD tick distribution in the affected region^15,16^. Seasonal outbreaks occur from December to May when the density of the nymphal stages of the tick is maximum^17^. Humans are the incidental hosts for the disease with an estimated yearly incidence of 400–500 cases; case fatality rates range between 2 and 10%^9,18^. However, larger outbreaks with more than 2500 annual cases have been reported in the past signifying its public health importance^19^.

Spatio-temporal prediction modeling is a key epidemiological tool that aids in situational awareness and early warning of events related to infectious disease outbreaks. Infectious disease models rely on routine active or passive surveillance reports and may include meteorologic, demographic, and/or geographic data associated with disease events to make predictions about the probability of an outbreak^20^. Ideally, prediction modes should incorporate all the important drivers associated with the disease occurrence based on reliable quantitative evidence. Additionally, the required information should be available in real-time to make rapid predictions and notify concerned stakeholders and authorities for timely decision-making. However, obtaining timely and accurate epidemiological data is challenging especially with emerging or neglected diseases, such as KFD.

Event-based surveillance (EBS) refers to the organized, rapid capture of information about potential health emergencies usually obtained from informal channels^21,22^. The information is gathered directly from hospitals, laboratories, news media, the internet, social media, and other informal sources without the involvement of government agencies and other traditional routes of reporting for faster detection of disease events. News media acts as an important means of information for EBS in most developed and developing countries. This data source has been successfully used for the early epidemiologic assessment of disease outbreaks, such as Rift Valley Fever^23^, Cholera^24^, Anthrax^23^, Ebola^25^, and COVID-19^26^. In addition, the internet has become an important source of health-related information for millions of people worldwide. Spikes in this information-seeking behavior have been shown to correlate with disease presence and have been monitored for real-time surveillance of diseases, such as influenza^27,28^, dengue^28,29^, and plague^30^. Since these EBS data are easy to access, they are being more routinely used by official health agencies, including World Health Organization, as a preliminary source of epidemiologic intelligence^31^.

Disease prediction models frequently require large amounts of quality epidemiological data for optimal performance. However, obtaining such datasets in real-world scenarios is most often difficult, expensive, and sometimes outright impossible, especially in the case of emerging or novel diseases. Modern machine learning (ML) approaches have addressed such limitations by using alternate sources of data as a starting point for model building. Transfer learning (TL) refers to a collection of techniques used to improve the predictions of one task by transferring information from a related task using ML. This transfer of knowledge, by extrapolating from pre-trained source models, facilitates target models to perform better with less training data and time than they typically would require^32,33^. The source task and target task are often closely related to, but not the same as, each other. The technique has been extensively used in many fields, including medical image-based diagnostics to diagnose conditions such as COVID-19^34,35^ and mpox^36^. In recent years, TL has shown promising results in the field of infectious disease predictions; forecasting capabilities of diseases with limited available data, such as dengue^37^, Zika^38,39^, and COVID-19^38,40,41^, were improved by supplementing source data either from closely related diseases and/or locations.

In this paper, we highlighted new time-series prediction strategies using KFD, an emerging zoonotic disease as an example. Our goal was to identify the best prediction models for situational awareness and forecasting of KFD cases at both the national and regional levels. We utilized EBS data along with climate data to increase the prediction performance of the models. Additionally, we tested TL techniques and their ability to increase the accuracy of KFD prediction in new outbreak locations by using data from an endemic region.

## Methodology

### Study area

Geographically, KFD is currently restricted to the Western Ghats of Southern India, which is considered one of the eight “hottest biodiversity hotspots”^42^. This region contains chains of mountains running approximately 1600 km in parallel with the western coast of India. KFD was first identified in 1957 within the Shimoga district of Karnataka and has since been endemic to the region. Nevertheless, in the past couple of decades, the disease has been gradually expanding to the neighboring southern state of Kerala and the northern states of Goa and Maharashtra.

## Data

### KFD case counts

For the ground truth data, we used monthly KFD cases reported by the country’s official health agency, the National Centre for Disease Control (NCDC), through its Integrated Disease Surveillance Program (IDSP) website^43^. However, the data provided by IDSP sometimes did not have case count information at a monthly granularity, which was essential for our prediction models. Furthermore, there was a possibility of not reporting or under-reporting KFD cases to the official health agency. To account for these inconsistencies, we conducted a systematic literature search following the Preferred Reporting Items for Systematic Reviews and Meta-Analyses (PRISMA) framework^44^ and looked for monthly KFD case reports in India. We specifically looked for the cases detected and reported by a designated KFD diagnostic laboratory. We used the search keywords “(Kyasanur forest disease OR Monkey fever) AND India” and searched scientific literature databases; Web of Science (webofscience.com), PubMed (pubmed.ncbi.nlm.nih.gov), and Google Scholar (scholar.google.com) and looked for case reports of KFD between 2010 and 2022. The monthly case counts along with outbreak-specific geographic information (i.e., state and/or district) reported in both IDSP and the scientific literature were documented and used as ground truth for our study. In case of discrepancy in reporting by two sources, the information available in the scientific literature was used.

### Weather data

The weather data was obtained from the National Aeronautics and Space Administration (NASA), POWER project website^45^. Temperature, humidity, and precipitation-related information were used as the input features for the time series prediction of KFD. The monthly averaged weather data for each district (spatial resolution of 55 × 55 km) that reported KFD outbreaks, either in IDSP or scientific literature between October 2010 and October 2019, was obtained.

### Event-based surveillance data

For this study, the open-source EBS data harvested from the internet included verified news reports and Google Trends.

News reports: ProMED is a large global infectious disease surveillance platform that reports health events related to emerging and re-emerging infectious diseases published by both official and verified unofficial sources^46^. The open-source KFD cases reported in India by local news media were gathered using ProMED. Each unofficial KFD event published in ProMED was identified and grouped based on its location and time of the outbreak. Using these reports, monthly unofficial KFD case count data for each region in India was created by manual extraction of information. This grouping of KFD news reports along with the manual extraction of information prevented the inclusion of case counts from multiple news articles reporting the same outbreak. To model real-life situations where official case counts are not immediately available, we used unofficial news report case count data as an input feature in our models.

Google Trends: Google Trends analyzes the popularity of search queries based on keyword search volume in Google Search across locations over time^47^. We obtained monthly Google Trends data for the keywords “Kyasanur forest disease” and “Monkey fever” at the national level and for each state that reported KFD between 2010 and 2019.

In this study, we built KFD models to predict the monthly distribution of KFD cases at the national and regional levels. Datasets at the regional and national spatial resolutions were created by averaging the weather across district-level data and by summing the unofficial cases identified from news data collected at the state level. Google Trends data was obtained at both the national and state-level resolutions and was directly used in their respective datasets when appropriate. For regions that spanned multiple states, the state-level Google Trends datasets were averaged together and rescaled back to the 0–100 range. The complete list of features used in our study is presented in Table 1.

**Table 1:** Description of the features used in KFD prediction models from Oct 2010 to Oct 2019.

Date type	Feature	Description
Weather	Specific humidity	Specific humidity at 2 meters (g/kg)
	Relative humidity	Relative humidity at 2 meters (%)
	Surface temperature	Earth skin temperature (C)
	Maximum temperature	Temperature at 2 meters maximum (C)
	Minimum temperature	Temperature at 2 meters minimum (C)
	Temperature range	Temperature at 2 meters range (C)
	Precipitation	Daily precipitation (mm/day)
EBS	News KFD cases	Unofficial KDF case counts harvested from local news reports
	News KFD deaths	Unofficial KDF death counts harvested from local news reports
	Monkey fever google trends	Google search trend for “Monkey fever”
	KFD google trends	Google search trend for “Kyasanur forest disease”

### Data preprocessing

**Figure 1:**
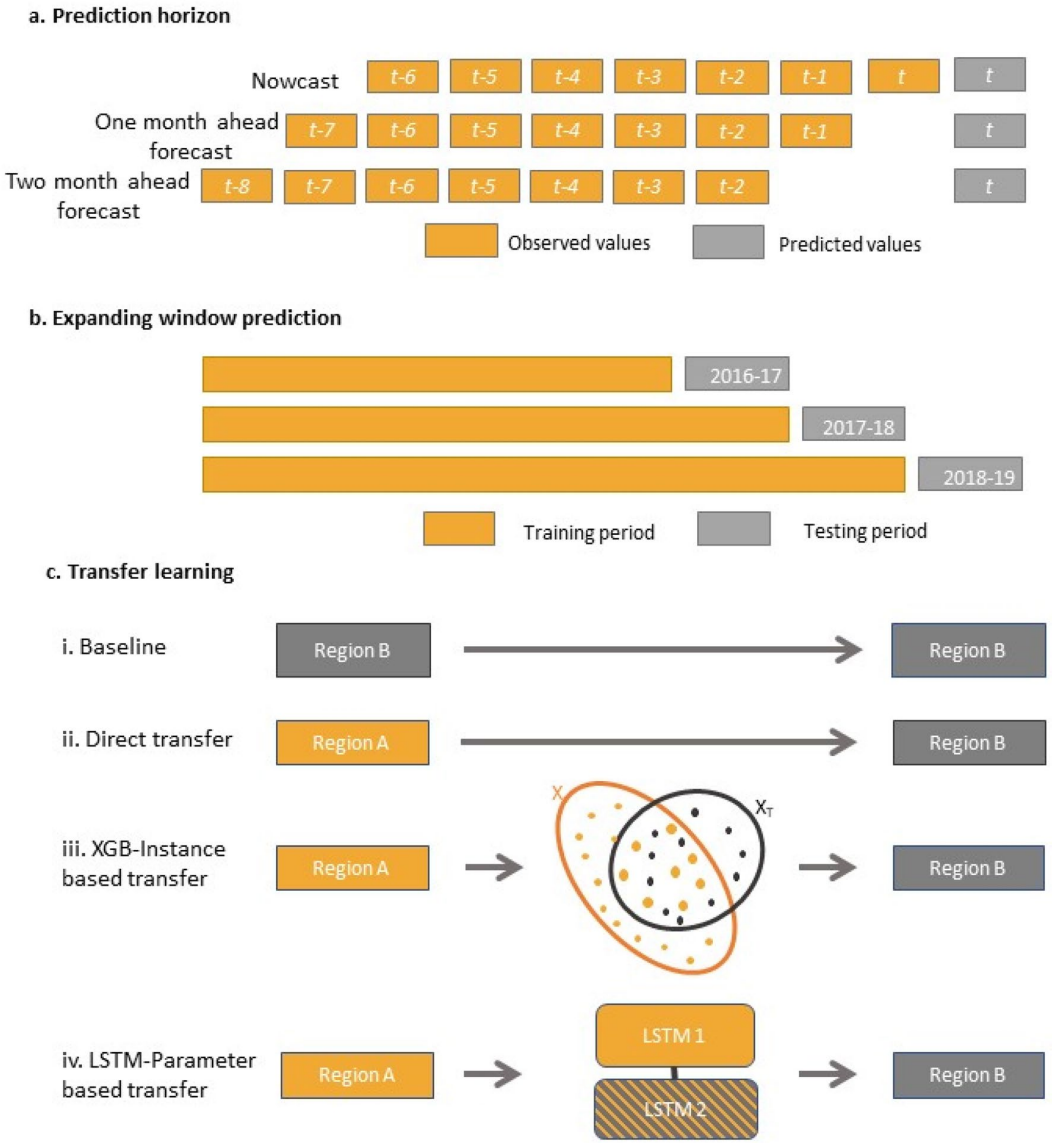
Schematic representation for KFD data preparation and TL: (**a**) Rolling window framework used to create time lags of input features for each prediction horizon: nowcast, one month ahead forecast, and two months ahead forecast; (**b**) Expanding window approach for yearly retraining of models between October 2016 to October 2019 (Note: The starting date of the model training depended on the year KFD was first reported in the location); and (**c**) Various TL techniques used for KFD regional-level prediction.

We conducted “rolling window” predictions where the most recent available feature data was used to predict the present or future KFD cases. For each month, our models generated predictions for three temporal horizons: (a) nowcast, (b) one month ahead forecast, and (c) two months ahead forecast (Fig. 1a). The nowcast model utilized features of the existing months to predict the current status of the disease. One and two months ahead forecasting models predicted the future using only the data available to them up to that month. This method ensured that we avoided look-ahead bias in our prediction by excluding information that would not have been available during model training in a real-life situation. The KFD’s transmission occurs during the drier and hotter months i.e., from November to May which is preceded by high precipitation from June to October^15,16^. Hence, each dataset contained features with up to a 6-month time series lag. For example, for the month *t*, the nowcast models utilized months *t* to *t-6* feature data points whereas 2-months ahead forecast models used months *t-2* to *t-8* feature data points (Fig. 1a). Furthermore, all the models predicted KFD cases for three years (October 2016 to October 2019) in an expanding window approach with the models periodically retraining at the beginning of the outbreak season (October) every year (Fig. 1b).

## KFD modeling

### Modeling techniques

#### National prediction

National-level data sets were created by aggregating feature datasets at the national level. Two separate models were built, namely a) the weather model that contained only weather data as input features and b) the weather and EBS model that contained weather, news reports, and Google Trends data as input features. Model performances were compared to determine the influence of each feature type on KFD predictions.

We used two different regression algorithms, Extreme Gradient Boosting (XGB) and Long Short-Term Memory (LSTM), for the prediction of KFD cases. These techniques are widely popular in temporal prediction tasks due to their ability to handle large and complex datasets and still result in high prediction accuracies^48,49^.

The XGB model is a tree-based ML technique that is an effective and scalable implementation of the gradient boosting framework^50^. This technique gradually and sequentially produces hundreds of low-accuracy models, called decision trees, and combines them into a single highly accurate prediction model. For our study, we used grid search with time series cross-validation split to optimize XGB models and identify the most accurate predictive hyperparameters. The permutation feature importance based on negative root mean square error (RMSE) was estimated to quantify the relevance of each feature in model performance.

The LSTM model is a deep learning architecture that is built upon the Recurrent Neural Network framework^51^. LSTMs can memorize the sequential data with long-term dependencies to accurately predict future values. This ability makes them an ideal candidate for time series prediction tasks involving long-term trends, including seasonal and cyclic variations. We built stacked LSTM models containing two LSTM layers, a drop-out, and a dense layer. Furthermore, we used grid search hyperparameter tuning with k-fold cross-validation split to ensure optimal model performance.

#### Regional prediction

KDF outbreak locations were divided into regions to obtain case count predictions at a higher spatial resolution. Region-wise divisions were made based on administrative (state or district level) borders shared between the outbreak location, spatial proximity, and the year the disease was first reported in the area. These criteria, in turn, determined the quantity of information available to train the prediction models. We used XGB and LSTM models as baseline models and applied TL approaches (i.e., direct transfer, fine-tuned instance-based transfer, and fine-tuned parameter-based transfer) as an extension of these models for the regional-level predictions.

The TL method enables sharing of KFD outbreak information between regions, which may improve the prediction performance of XGB and LSTM models when available data is limited for specific regions. The following models were constructed at the regional level.

- Baseline: Baseline models were built by training and testing on a single region; there was no transfer of knowledge between the regions in these models. These models provided a benchmark to compare the performances of TL models. Baseline models were created for both XGB and LSTM approaches (Fig. 1c).
- Direct transfer: For direct transfer, the models were trained on the source region, which was directly used to predict KFD cases in the target region without further retraining. This transfer technique was applied to both XGB and LSTM models (Fig. 1c).
- Finetuned transfer: Finetuning refers to training the model with source region data followed by reallocating model weights by partially retraining it with the target region data. In our study, finetuning was performed by the following methods:
  i. Instance-based transfer: This method was applied to XGB models utilizing TrAdaBoost.R2 algorithm^52^. This technique is based on the principle of “reverse boosting” wherein the weights of source instances are reduced at each boosting iteration for every poor prediction made whereas the weights of target instances are increased. In our case, the observations of source regions that are different from the target region receive lesser weights and, therefore, have a reduced influence on model prediction. We applied TrAdaBoost.R2 algorithm with XGB for 20 iterations at a learning rate of 0.01 (Fig. 1c).
  ii. Parameter-based transfer: This method was applied to LSTM models. Parameter-based finetuning facilitates the parameter transfer where the model utilizes the weights generated by both source and target region datasets to improve the predictive performance of the target region. The model was first trained on the source region. For TL, the first layer of the pre-trained model was considered non-trainable. The remaining layers were unfrozen, and the model weights were updated by retraining with the target region data. Retraining was carried out for 50 epochs with a learning rate of 0.005 (Fig. 1c).

For national-level models, the training period was between Oct 2010 to Sept 2016. For the regional level, the training data varied by region based on the first observed outbreak within the study period. The starting date of the training data was Oct 2010 for Karnataka, Oct 2012 for Kerala and Oct 2014 for Goa and Maharashtra. The testing period was between Oct 2016 to Oct 2019.

### Uncertainty estimation

We calculated prediction uncertainties for the best-performing models. The 95% prediction intervals were calculated using the bootstrap technique. This nonparametric approach computes prediction intervals with no specific assumptions about the sampling distribution of the noise or the data^53^.

### Model evaluation

Mean Absolute Error (MAE) and RMSE were used to evaluate and compare model performances. The MAE is the average absolute error between the predicted and real values whereas RMSE is the square root of the mean of the square of the error.

## Results

### KFD cases

**Figure 2:**
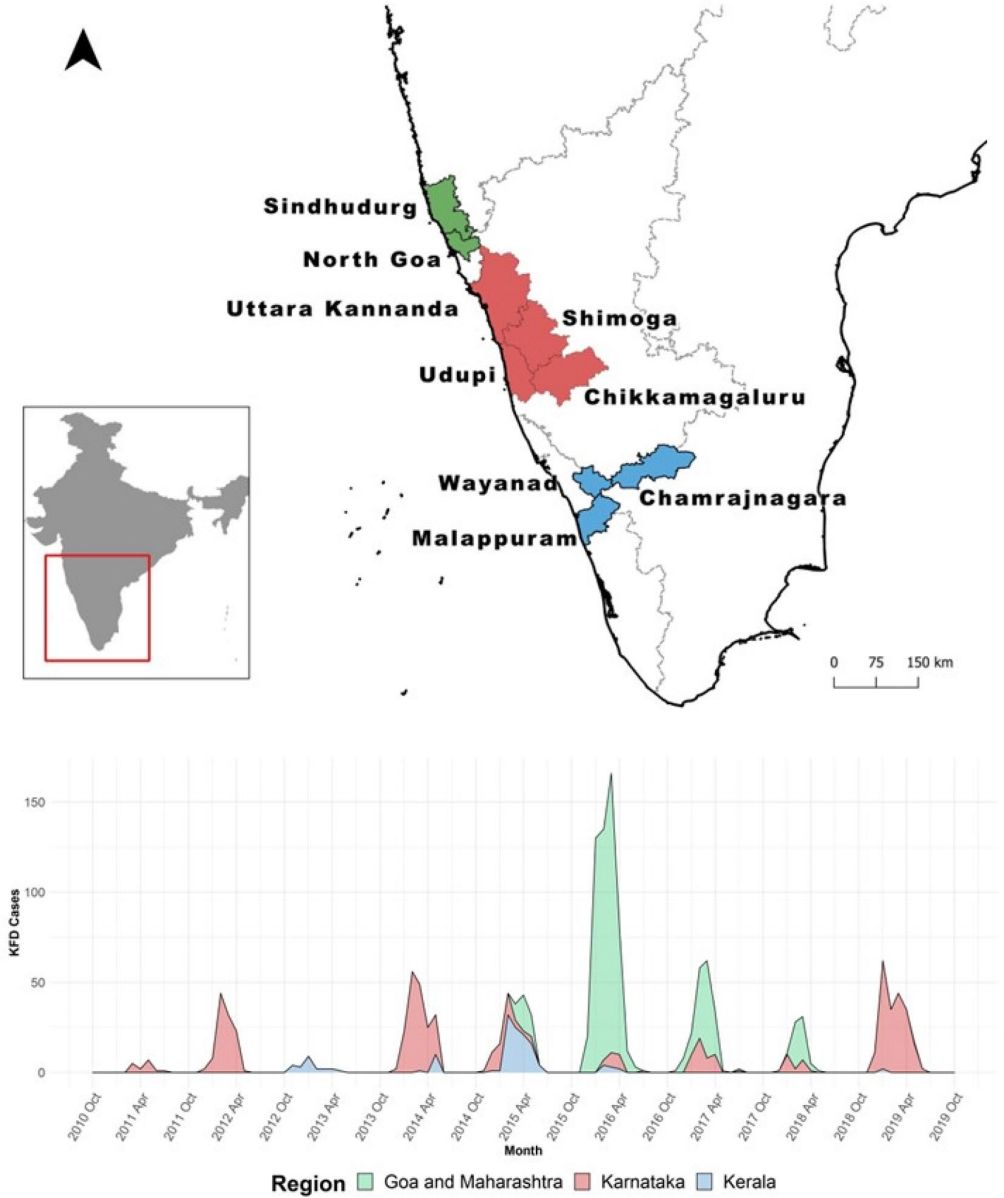
Spatiotemporal distribution of KFD in India (Oct 2010-Oct 2019): Region and district-wise breakdown of KFD outbreak locations (top) along with the time series distribution (stacked area graph) of monthly KFD cases in affected regions (bottom). The green, red, and blue colors represent Goa and Maharashtra, Karnataka, and Kerala regions, respectively. The map was generated using QGIS 3.28.8 software (www.qgis.org).* Chamarajanagar district is present in Karnataka state. However, it was grouped in the Kerala region due to the temporal and spatial proximity of the outbreak with other outbreaks in the region.

We searched the IDSP website and found a total of 15 KFD outbreak events reported between Oct 2010 and Oct 2019 (Supplementary Table S1). The average difference between the start of an outbreak and reporting date was 26.7 days and a maximum of 91 days. The systematic literature search of scientific databases yielded 4132 articles (PubMed: 3187, Google Scholar: 200, Web of Sciences: 115). Among these, 10 publications had details of monthly KDF case counts at the state level or higher resolution (Supplementary Table S2). All published KFD cases were detected in a designated KFD diagnostic laboratory. For our study, Shimoga, Uttara Kannada, Chikkmagaluru, and Udupi districts of Karnataka State, which have been historically considered endemic areas for the disease since it was first discovered in 1957^3^, were grouped as Karnataka region. Chamarajanagara district of Karnataka and neighboring Wayanad and Malappuram districts of Kerala encountered their first KFD outbreaks between 2012 and 2014^4–6^. These districts are towards the south of the endemic region and hence were grouped as the Kerala region. Finally, the North Goa district of Goa and Sindhudurg district of Maharashtra had their first disease outbreaks in the year 2015 and 2016, respectively^7,8^. These districts are towards the north of the endemic region and were grouped as Goa and Maharashtra region. We documented monthly case counts of KFD at the defined regional level and aggregated this information to get country-level monthly KFD cases. The spatiotemporal distribution of KFD cases between October 2010 and October 2019 in India is presented in Fig. 2.

For ground truth data, we found only a total of 520 cases of KFD reported between October 2010 and October 2019 on the IDSP website. However, KFD case counts increased to 1545 when reports from PRISMA systematic literature were considered along with IDSP reports suggesting a considerable underreporting of KFD by official health agencies. This underreporting was observed in all the regions but was more pronounced in Karnataka and Kerala (Supplementary Table S3).

## National prediction

**Figure 3:**
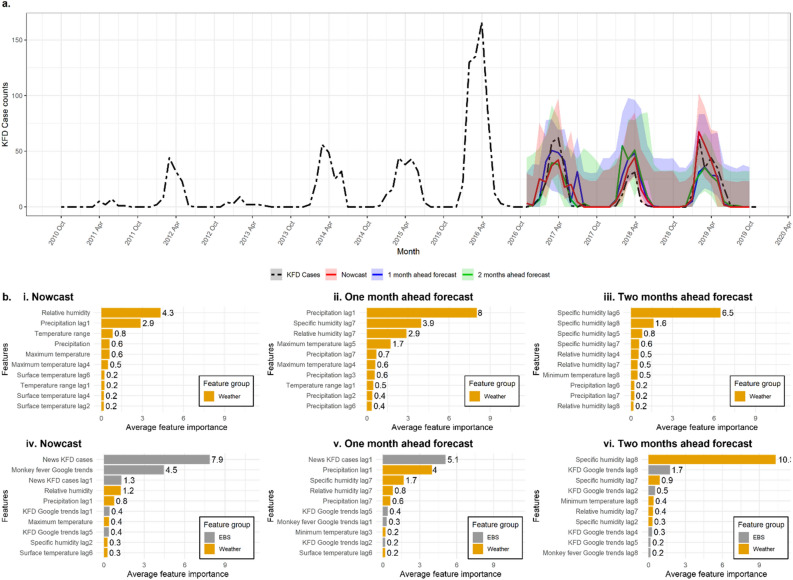
The KFD monthly predictions in India. (**a**) The time series results of the best-performing technique, XGB-weather and EBS model (Oct 2016—2019). The shaded areas represent 95% prediction intervals for the respective prediction horizons. (**b**) The top 10 features for XGB models based on average permutation feature importance (negative RMSE scores) for nowcasts, 1-month ahead, and 2-months ahead forecasts for weather-only (top) and weather plus EBS models (bottom).

Based on RMSE and MAE values, the models with weather and EBS features outperformed weather-only models across different prediction horizons and model types. The nowcasts had the least prediction error compared to 1-month and 2-months ahead forecasts. The XGB models performed better compared to LSTM (Table 2). The overall best-performing time series models per prediction horizon, i.e., XGB with weather and EBS, are presented in Fig. 3a.

**Table 2:** The error metrics of the baseline national level prediction models across prediction horizons and included feature types (Oct 2016—Oct 2019).

Feature type	Prediction horizon	XGB		LSTM	
		RMSE	MAE	RMSE	MAE
Weather	Nowcast	10.8	5.9	17.1	11.0
	1-month ahead forecast	14.4	8.1	14.3	9.0
	2-months ahead forecast	12.8	7.6	15.3	8.5
Weather and EBS	Nowcast	**9.1**	**5.8**	12.0	7.8
	1-month ahead forecast	**11.0**	**6.7**	14.1	8.0
	2-months ahead forecast	**12.8**	**7.2**	12.7	7.6

The bolded values represent the models with the smallest RMSE and MAE.

We estimated the average permutation feature importance of XGB models for all three prediction years (Oct 2016—Oct 2019) (Fig. 3b). For weather-only models, humidity and precipitation-related features were most important for KFD predictions for all prediction horizons. On the other hand, for weather plus EBS models, the dependence on EBS variables decreased whereas the dependence on weather variables increased with the increasing forecasting horizon. For nowcasts, internet-related features, including news reports and “monkey fever” Google search trends, were the top two most important features. News reports and precipitation-related features were almost equally important for 1-month ahead forecasts whereas the humidity-related features were the most important for 2-months ahead forecasts.

## Regional prediction

For regional models, we first created baseline models where only the target regional-level data was used for both training and testing. For non-endemic regions, we also built TL models using two techniques, i.e., direct transfer and fine-tuned transfer, with the aim to increase KFD predictive capabilities for each region. All the models were trained using climate and EBS data. The list of source and target region datasets used for training TL models is presented in Table 3.

**Table 3:** The source and target datasets used for training regional-level TL models.

Target region	Source region	
	Direct transfer	Fine-tuned transfer
Karnataka	–	–
Kerala	Karnataka	Karnataka
Goa and Maharashtra	Karnataka and Kerala	Karnataka and Kerala

The predictive performances of all the models measured using RMSE and MAE values at the regional level are shown in Table 4. Karnataka, the endemic region for KDF, constantly reported outbreaks throughout the study duration except for 2012–2013. Due to disease endemicity, we only built baseline prediction models for this region. The predictive errors of the models increased with increasing predictive horizon. Overall, all the models correctly predicted this low KFD case trend in Kerala; the XGB-direct transfer model performed slightly better than the rest. Goa and Maharashtra, the most recent disease foci, had the least amount of time series data to train the model. Both direct and fine-tuned TL models outperformed baseline ones with LSTM fine-tuned having the smallest prediction errors. Furthermore, the overall model errors increased with the increasing prediction horizon.

**Table 4:** The performance metrics of the KFD prediction models at the regional level. The bolded values represent the models with the smallest RMSE and MAE across modeling techniques and prediction horizons for each region.

Region	Transfer Learning	Prediction horizon	XGB		LSTM	
			**RMSE**	**MAE**	**RMSE**	**MAE**
**Karnataka**	Baseline	Nowcast	**7.8**	**4.2**	7.9	4.4
		1 month ahead forecast	**12.6**	**5.7**	13.6	6.2
		2 months ahead forecast	**15.0**	**6.9**	15.2	6.9
**Kerala**	Baseline	Nowcast	0.7	0.3	2.6	1.8
		1 month ahead forecast	1.2	0.7	4.2	2.8
		2 months ahead forecast	1.6	1.1	2.7	2.0
	Direct transfer	Nowcast	**0.4**	**0.1**	1.1	0.7
		1 month ahead forecast	**0.6**	**0.2**	1.4	0.9
		2 months ahead forecast	**0.5**	**0.1**	1.5	1.2
	Finetuned transfer	Nowcast	0.4	0.1	2.4	1.3
		1 month ahead forecast	0.7	0.3	1.7	1.0
		2 months ahead forecast	0.8	0.4	2.8	1.2
**Goa and Maharashtra**	Baseline	Nowcast	14.6	8.2	16.1	9.1
		1 month ahead forecast	14.8	7.9	24.2	13.6
		2 months ahead forecast	10.6	7.0	11.4	7.1
	Direct transfer	Nowcast	10.4	5.6	10.5	5.4
		1 month ahead forecast	11.5	6.7	11.5	6.1
		2 months ahead forecast	12.4	5.0	10.8	5.9
	Fine-tuned transfer	Nowcast	12.4	5.9	**7.0**	**3.7**
		1 month ahead forecast	15.4	8.3	**9.3**	**4.9**
		2 months ahead forecast	**10.1**	**5.6**	11.1	5.1

**Figure 4:**
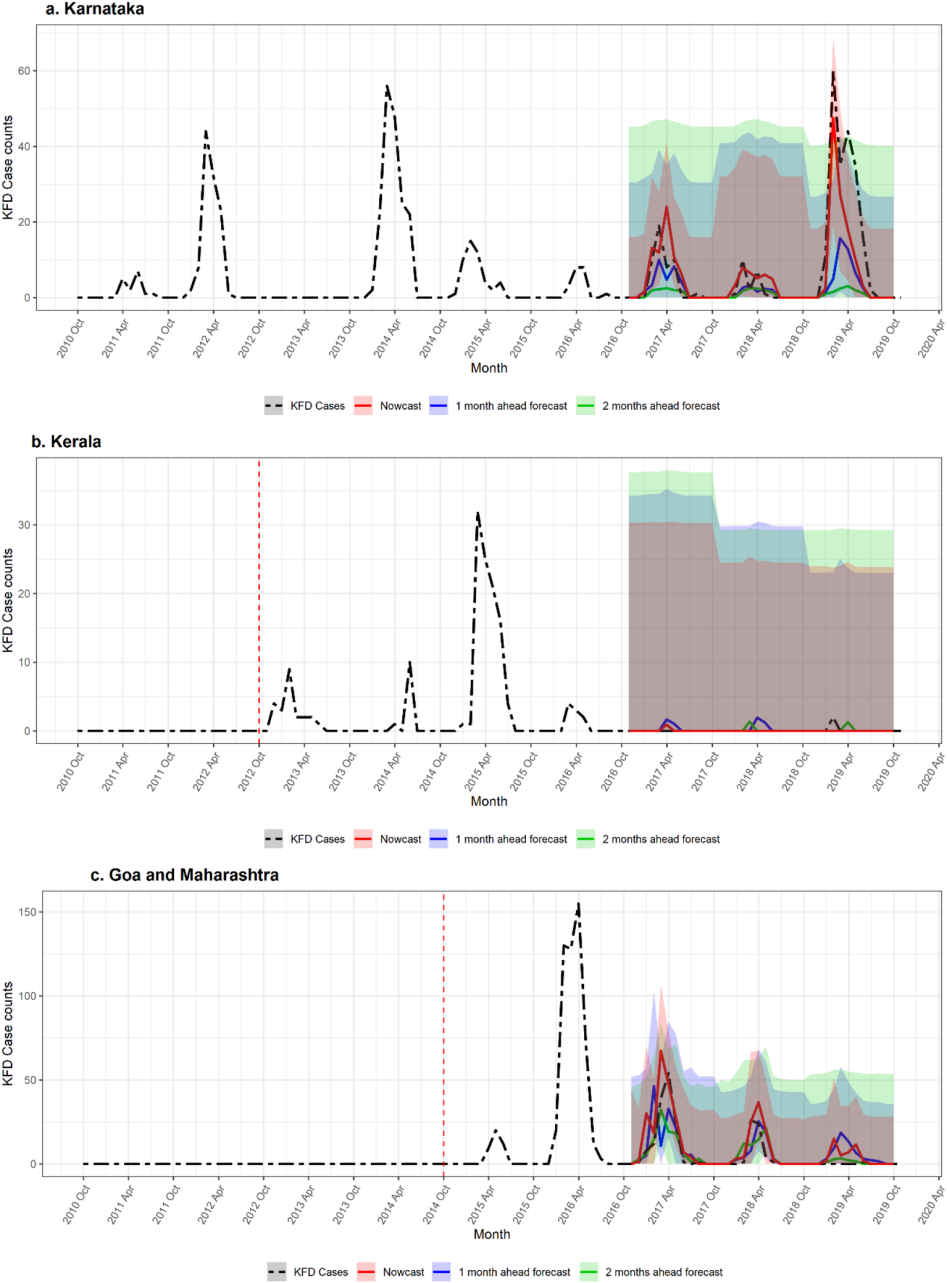
Regional-level KFD monthly predictions in India. The time series result of the best performing XGB models, (**a**) Karnataka: baseline model, (**b**) Kerala: direct transfer model, (**c**) Goa and Maharashtra: direct transfer model. The vertical dotted line indicates the first outbreak season in the region. The shaded areas represent 95% prediction intervals for the respective prediction horizons.

Overall, the XGB models had better predictive performance compared to LSTM across regions and predictive horizons. Time series prediction graphs from those best-performing XGB models for each region are presented in Fig. 4. In all locations, the width of the 95% prediction interval (PI) increased as the prediction horizon grew. For the Karnataka base model, the 95% PI width decreased as predicted case counts increased. In contrast, transfer learning models for Kerala, Goa and Maharashtra had a consistent 95% PI width regardless of the predicted case counts.

## Discussion

Prediction models could provide crucial epidemiological foresight about disease outbreaks for better risk assessment and implementation of necessary interventions in the affected population. However, building modeling capabilities applicable to real-world scenarios is often a challenge due to the limited availability of quality and timely data. These limitations are compounded when trying to predict diseases in new geographic foci and/or where disease surveillance and public health infrastructure are not well established. This KFD study is the first to demonstrate the merits of combining open-source unofficial case count data from local news and internet searches with traditional climate data to strengthen the predictive capabilities of a disease facing all of the above challenges. Forecasting models rely heavily on historic data represented as time lags to improve predictive accuracies^48,49^. Our study showed that unofficial case counts reported in news media could be used as an alternative to historic data obtained from official sources to produce timely and reliable nowcasting and short-term forecasting estimates. This study is also the first to generate KFD time series prediction models, both at the national and regional levels, using advanced ML algorithms and TL techniques.

In India, disease surveillance is overseen at the central (federal) government level. The IDSP under the NCDC, Ministry of Health and Family Welfare maintains decentralized laboratory-based disease surveillance systems at the state and district level to track, detect, and respond to health emergencies^43^. The IDSP has a portal for weekly reporting of important infectious disease outbreaks across India. This surveillance includes KFD detection and reporting through the Viral Diagnostic Laboratories (VDL), which are the designated laboratories for processing KFD samples at the state or regional level^54^. A confirmed KFD case in humans is followed by mass vaccination within 5 km radius of the infected zone and sero-surveillance efforts as primary control measures^55^. However, we found a considerable underreporting and delayed reporting of KFD events by official agencies. Furthermore, weekly updates on the status of outbreaks, such as disease progression and applied interventions, were sometimes missing. Our finding is consistent with another study in Karnataka that found similar reporting delays and data quality issues; That study concluded that the KFD surveillance conducted at the district and state level might not have fully integrated with the national IDSP portal system^56^. Moreover, KFD case reporting systems were not maintained by health authorities at the state or district level. To account for the discrepancies in KFD cases diagnosed locally and the cases reported to the national health authorities, we conducted a systematic literature review and looked for monthly KFD case reports that were diagnosed at VDLs. Including both IDSP and systematic review case counts ensured an extra level of completeness in the data and accounted for reporting bias in our prediction models.

Our results showed that including EBS data along with weather data increased the predictive performance across all models and prediction horizons in comparison with weather-only models at the national level. Hence, EBS data, such as online news articles and Google trends, could be an important source of information to improve nowcasting and short-term (1 or 2 months) forecasting performances of KFD models. With rapidly growing internet usage in remote locations and rising health literacy among populations, these non-traditional means of disease intelligence have become more readily available and a vital source of data for zoonotic disease prediction under resource and data-limited settings^23^. This current study clearly demonstrates how the inclusion of internet data along with traditional climate data can improve the performance of forecasting models. The local news media strives to report real-time disease outbreaks and other health-related information gathered directly from local administrative agencies, public health institutes, hospitals, and other lower organizations. This form of information dissemination plays an important role in risk communication for the general public. Due to a lack of strong collaborations and communication chains between many regional and central health agencies, disease outbreaks are frequently bought to the attention of the more centralized health authorities through the media sensationalizing the situation^57^. On the other hand, the internet is also growing as an important source of information during public health crises across the globe^58,59^. Google trends represent the interest of the general public in a particular topic at a given time. During a disease outbreak, google trends can be used as an additional biosurveillance tool along with the traditional disease surveillance strategies by tracking disease-related keyword searches on the internet^60^. Historically, KFD outbreaks have exhibited temporal inconsistencies with a few focal periods of large outbreaks followed by relatively less severe years^19^. These variations are unable to be explained and therefore predicted, by meteorological variables alone. A public health emergency can quickly lead to early news media coverage, sometimes sensationalizing the event before medical reporting occurs^61,62^. The media coverage results in the public turning to the internet and social media for more public health information. This initial community response might be the reason for high short-term prediction accuracies for our KFD models, which were not as remarkable as the prediction horizon increased.

Care should be taken when using EBS data in disease prediction. Firstly, EBS data alone might lack the sensitivity to recognize disease presence, especially for neglected zoonotic diseases whose overall disease burden remains at a fairly constant endemic threshold^23^. These diseases are often underrepresented in research interest, government policies, and public awareness. Hence, the application of EBS alone may not be ideal to monitor such diseases. Conversely, some disease events, such as those with epidemic or pandemic potential, could get overrepresented by the news media which results in increased reporting and public engagement^57^. Such exaggerations may result in the overfitting of time-series models and subsequently an overestimation of the disease burden. Misrepresentation or disinformation of EBS data can result in either underrepresentation or overrepresentation of disease presence also leading to inaccurate forecasts. Taking a One Health approach, i.e., including other important drivers related to the disease ecology along with the EBS data, and including frequent retraining of the time-series models can help minimize these biases.Forecasting endemic zoonotic disease is a difficult task especially when the factors responsible for the temporal and spatial variations in disease outbreaks are unclear. The challenges are compounded when the effectiveness of public health decisions, such as the application of control and prevention measures, in the affected region are varied over time and location. Including such intervention-related information along with other ecological determinants could help increase the performance of the disease forecasting models. Building such predictive capabilities would require real-time data and infrastructure sharing between concerned agencies and stakeholders from a One Health perspective both at the national and regional levels. Such critical collaborations are currently lacking in India^63^.

We used TL techniques and compared them with baseline models to evaluate the strengths of incorporating existing outbreak-related knowledge from an endemic region into models of new disease foci. Overall, the short-term KFD predictive performance in Kerala, Goa, and Maharashtra increased when TL was implemented. The difference was more evident in Goa and Maharashtra where the baseline models had very limited data to train with. Furthermore, the strong performance of the direct transfer models is notable even though they did not use any information related to the target regions during model training. Our results are consistent with previous studies that utilized the direct transfer method^38,39^. This technique could be valuable for situational awareness and preparedness for new and emerging disease foci where available epidemiological information is scarce. Alternatively, the method could also be useful in areas where disease surveillance systems are rudimentary or deteriorating due to a lack of robust public health infrastructure. The strong performance of the direct transfer models could be attributed to the geographic, climatic, and demographic similarities between KFD outbreak locations, resulting in a more accurate transfer of knowledge from one region to another^6,15,64^. Nevertheless, care should be taken when using model extrapolation for decision-making since the prediction accuracy of the TL methods found here could be a result of the disease and locations used in this study.

There are some limitations to our study. First, we did not consider small sporadic KFD cases that occurred outside the three outbreak regions, regardless of when they took place. We specifically chose the target regions in our study based on their close spatial locations with outbreaks occurring during a similar time period. Second, we were conservative with the search terms used for our Google Trends data and queried only selected keywords in English, which might have excluded some important patterns for prediction models. We did not include Google keyword search terms in the regional languages. However, initial results of news articles and Google search keywords in the regional languages produced very limited results. News reports in regional languages play an important role in the timely dissemination of public health information to the local, general public. Including data extracted from local print media could improve disease surveillance efforts in the future.

In conclusion, our study addressed the limitations of emerging zoonotic disease prediction when the available epidemiological information is limited, using KFD as a case study. Our study showed that combining open-source news reports and Google Trends data along with climate data strengthens the predictive capabilities of KFD time series models, both at a national and regional level. Furthermore, TL was effective in predicting the disease outbreaks in new disease foci i.e., Kerala, Goa, and Maharashtra, by using data from Karnataka, where KFD is endemic. These ML capabilities could be generalized to predict outbreaks into the future and in new locations. Especially when outbreak-related information is delayed, the prediction methods used here would be highly advantageous to public health authorities enabling better situational awareness and preparedness. Regardless, to be able to mitigate KFD from continuing to spread and become endemic in new areas, there is an urgent need for better data-sharing between governmental health agencies and other stakeholders, both at the local and national levels in a One Health context, to strengthen disease surveillance and public health infrastructure for improved decision-making in India.

## Supplementary Information

Supplementary Information.

## Data Availability

The datasets presented in this study are available on request from the corresponding author.
