# Supplementary material for: Predicting Kyasanur forest disease in resource-limited settings using event-based surveillance and transfer learning
Source: Sci Rep. 2023 Jul 8;13:11067. doi: 10.1038/s41598-023-38074-0 (PMC10329696; doi:10.1038/s41598-023-38074-0)
Supplement: Supplementary file 1 — Supplementary Information. [file 41598_2023_38074_MOESM1_ESM.docx]

**SUPPLEMENTARY INFORMATION**

**Predicting Kyasanur Forest Disease in resource-limited settings using event-based surveillance and transfer learning**

Ravikiran Keshavamurthy^1,2^ and Lauren E. Charles^1,2^ *

^1^Pacific Northwest National Laboratory, Richland, WA 99354, USA

^2^Paul G. Allen School for Global Health, Washington State University, Pullman, WA 99164, USA

*Correspondence: [lauren.charles@pnnl.gov](mailto:lauren.charles@pnnl.gov)

**Supplementary Table S1: Details of KFD events that were reported on the Integrated Disease Surveillance Program (IDSP) website between October 2010 and October 2019**

| **State** | **District** | **Cases** | **Deaths** | **Start of outbreak**  **(dd/mm/yyyy)** | **Date of**  **reporting to IDSP**  **(dd/mm/yyyy)** | **Difference between start and reporting dates of outbreak** |
| --- | --- | --- | --- | --- | --- | --- |
| Karnataka | Uttar Kannada | 2 | 1 | 02/04/2011 | 21/04/2011 | 19 |
| Karnataka | Shimoga | 38 | 1 | 23/12/2011 | 12/02/2012 | 51 |
| Goa | North Goa | 14 | 0 | 13/12/2015 | 03/01/2016 | 21 |
| Maharashtra | Sindhudurg | 13 | 0 | 13/01/2016 | 18/01/16 | 5 |
| Maharashtra | Sindhudurg | 242 | 1 | 13/01/2016 | 13/03/2016 | 59 |
| Goa | North Goa | 154 | 1 | 13/12/2015 | 13/03/2016 | 91 |
| Goa | North Goa | 1 | 0 | 04/11/2016 | 08/11/2016 | 4 |
| Goa | North Goa | 1 | 0 | 05/12/2016 | 12/12/2016 | 7 |
| Goa | North Goa | 5 | 0 | 05/12/2016 | 25/12/2016 | 20 |
| Maharashtra | Sindhudurg | 20 | 1 | 19/01/2017 | 12/02/2017 | 24 |
| Maharashtra | Sindhudurg | 5 | 0 | 15/01/2018 | 11/02/2018 | 27 |
| Maharashtra | Sindhudurg | 5 | 2 | 11/03/2018 | 25/03/2018 | 14 |
| Karnataka | Shivamogga | 17 | 6 | 14/12/2018 | 06/01/2019 | 23 |
| Kerala | Wayanad | 2 | 0 | 23/01/2019 | 27/01/2019 | 4 |
| Maharashtra | Sindhudurg | 1 | 1 | 29/04/2019 | 30/04/2019 | 1 |

**Supplementary Table S2: The details of citations that were identified in the systematic literature search and included to create KFD** **case count data along with the regional and district level breakdown of KFD outbreak locations**

| **Region** | **Outbreak districts** | **Citation** |
| --- | --- | --- |
| Karnataka | Shimoga, Uttara Kannada, Chikkmagaluru, Udupi | ^1–5^ |
| Kerala | Chamarajanagara*, Wayanad, Malappuram | ^3,6–8^ |
| Goa and Maharashtra | North Goa, Sindhudurg | ^3,9,10^ |

* Chamarajanagar district is present in Karnataka state. However, it was grouped in the Kerala region due to the temporal and spatial proximity of the outbreak with other outbreaks in the region

**Supplementary Table S3: KFD case count data reported in the IDSP website and scientific literature between October 2010 and October 2019 in different KFD outbreak regions of India**

| **Year** | **IDSP** | | | **IDSP + scientific literature** | | |
| --- | --- | --- | --- | --- | --- | --- |
|  | **Karnataka** | **Kerala** | **Goa and Maharashtra** | **Karnataka** | **Kerala** | **Goa and Maharashtra** |
| 2010-11 | 2 | 0 | 0 | 16 | 0 | 0 |
| 2011-12 | 38 | 0 | 0 | 110 | 0 | 0 |
| 2012-13 | 0 | 0 | 0 | 0 | 23 | 0 |
| 2013-14 | 0 | 0 | 0 | 176 | 12 | 0 |
| 2014-15 | 0 | 0 | 0 | 48 | 100 | 41 |
| 2015-16 | 0 | 0 | 423 | 20 | 9 | 519 |
| 2016-17 | 0 | 0 | 27 | 48 | 0 | 140 |
| 2017-18 | 0 | 0 | 10 | 21 | 0 | 56 |
| 2018-19 | 17 | 2 | 1 | 203 | 2 | 1 |
| **Total** | **57** | **2** | **461** | **642** | **146** | **757** |

**References**

1. Gupta, N., Chunduru, K., Safeer K, M. & Saravu, K. Clinical and laboratory profile of patients with Kyasanur forest disease: A single-centre study of 192 patients from Karnataka, India. *Journal of Clinical Virology* **135**, 104735 (2021).

2. Kiran, S. K. *et al.* Kyasanur Forest Disease Outbreak and Vaccination Strategy, Shimoga District, India, 2013–2014. *Emerg Infect Dis* **21**, 146 (2015).

3. Singh, P., Kumar, P. & Dhiman, R. Kyasanur forest disease and climatic attributes in India. *J Vector Borne Dis* **59**, 79–85 (2022).

4. Thippeswamy, N. B. & Kiran, S. K. Outbreak of Kyasanur Forest Disease in Shivamogga, Karnataka State, India, during 2015. (2017). doi:10.1016/j.vetmic.2009.08.024.

5. Kasabi, G. S. *et al*. Kyasanur forest disease, India, 2011–2012. *Emerging infectious diseases* 19, 278 (2013).

6. Tandale, B. V., Balakrishnan, A., Yadav, P. D., Marja, N. & Mourya, D. T. New focus of Kyasanur Forest disease virus activity in a tribal area in Kerala, India, 2014. *Infect Dis Poverty* **4**, (2015).

7. Sadanandane, C. *et al.* An outbreak of Kyasanur forest disease in the Wayanad and Malappuram districts of Kerala, India. *Ticks Tick Borne Dis* **8**, 25–30 (2017).

8. Mourya, D. T., Yadav, P. D., Sandhya, V. K. & Reddy, S. Spread of Kyasanur Forest Disease, Bandipur Tiger Reserve, India, 2012–2013. *Emerg Infect Dis* **19**, 1540 (2013).

9. Oliveira, A. *et al.* Geospatial clustering, seasonal trend and forecasting of Kyasanur Forest Disease in the state of Goa, India, 2015-2018. *Trop Med Health* **48**, (2020).

10. Gurav, Y. K. *et al.* Kyasanur forest disease prevalence in western ghats proven and confirmed by recent outbreak in Maharashtra, India, 2016. *liebertpub.com* **18**, 164–172 (2018).
